# IRF2 Destabilizes Oncogenic KPNA2 to Modulate the Development of Osteosarcoma

**DOI:** 10.1155/2022/9973519

**Published:** 2022-09-26

**Authors:** Shuchi Xia, Yiqun Ma

**Affiliations:** ^1^Department of Dentistry, Zhongshan Hospital, Fudan University, Shanghai 200032, China; ^2^Department of Orthopedics, Zhongshan Hospital, Fudan University, Shanghai 200032, China

## Abstract

Osteosarcomas (OS) are the most common primary malignant bone tumor. Emerging evidence revealed that karyopherin alpha 2 (KPNA2) was strongly associated with the tumorigenesis and development of numerous human cancers. The aim of the present study was to investigate the expression pattern, biological functions, and underlying mechanism of KPNA2 in OS. Bioinformatics TFBIND online was applied to forecast transcription factor (TF) binding sites in the promoter region of KPNA2. The expression profile of KPNA2 in OS tissues were firstly assessed. CCK8, colony formation, wound healing, and Transwell assays were used to assess cell viability, proliferation, and migration in vitro, and in vivo experiments were performed to explore the effects of KPNA2 and interferon regulatory factor-2 (IRF2) on tumor growth. Furthermore, the correlation between IRF2 and KPNA2 was investigated using chromatin immunoprecipitation (ChIP), RT-qPCR, western blot, and dual-luciferase assays. KPNA2 was obviously upregulated, while IRF2 decreased significantly in OS tissues and cell lines, as well as negatively correlated with each other. KPNA2 removal remarkably suppressed OS cell growth, migration, invasion in vitro, and tumor growth in vivo, while IRF2 knockdown exerts an opposing effect. IRF2 binds to the KPNA2 promoter to modulate the malignant phenotypes of OS cells by regulating epithelial-to-mesenchymal transition (EMT). The present study demonstrated that KPNA2 performed the oncogenic function, possibly regulating tumor development through EMT. Importantly, it was confirmed that IRF2 serves as a potential upstream TF of KPNA2 involved in the regulation of EMT progress in OS.

## 1. Introduction

Osteosarcoma (OS) is the frequent primary malignant bone tumor, affecting mainly pediatric and adolescents [1], which is composed of malignant mesenchymal cells producing osteoid and/or immature bone [2]. It typically forms in the metaphysis of long bones, specifically the proximal tibia, the distal femur, and the proximal humerus, accompanied by swelling and pain [3]. It is common that metastasis to the lungs of OS [4]. Before the use of neoadjuvant and adjuvant chemotherapy, approximately 90% of OS patients died from lung metastases [5]. OS is characterized by high levels of genomic instability. However, the molecular basis involved in OS remains unclear and continuing to seek new treatments is urgently needed to continue looking for new treatments.

Karyopherin alpha 2 (KPNA2, 58 kDa) is one of seven members of the karyopherin *α*-family [6, 7]; Dysregulation of KPNA2 had been reported serving as a potential biomarker in several malignancies, including breast cancer [8], gastric cancer [9], lung cancer [10], and glioma [11]. KPNA2 served as the adaptor to transfer p65 to the nucleus to identify the classic nuclear localization signal [7, 12]. Emerging data suggest a role for the epithelial-mesenchymal transition (EMT) in the regulation of cellular plasticity in normal adult tissues and tumors, where they can generate multiple and distinct cellular subpopulations that contribute to intratumoural heterogeneity [13]. EMT is important for invasion, metastasis, and drug-resistance of cancer cells. A previous study showed that KPNA2 silence may reduce ovarian carcinoma migration and invasion by inhibiting Akt/GSK-3*β*/Snail pathway and suppressing EMT [14]. In addition, KPNA2 was involved in the regulation of autophagy and the EMT of glioblastoma cells [15]. However, the molecular pathways regulated by KPNA2-mediated EMT in OS are yet to be elucidated.

Transcription factors (TFs), could specifically recognize DNA through consensus sequences, thus to control chromatin and transcription, guiding expression of the genome [16]. To identify the TF of KPNA2 transcription, the online software of TFBIND (http://tfbind.hgc.jp/) was employed to identify putative binding sites in the promoter region of KPNA2. Specifically, several canonical IRF2-binding sites in the promoter region of KPNA2 were observed, and IRF2 was then extracted from 147 candidate genes. IRF2, belonging to the IRF family, was widely expressed in various tissues [17]. Among several types of cancer, belong to the IRF family, IRF1 signaling pathways may directly induce p21-dependent G0/G1 cell cycle and p21-independent modulation of survival [18]. IRF2 was shown to serve as an important regulator in acute myeloid leukemia by targeting INPP4B [19]. A recent study has indicated that IRF2 was able to suppress the strengthening of cell migration and invasion in OS, which were mediated by miR-18a-5p [20]. Similarly, *IRF2* was not expressed or at a low level in OS tissues [21]. Intriguingly, IRF was already identified as a functional TF in nonsmall cell lung cancer (NSCLC) that suppressed KPNA2 expression [22]. Therefore, we speculate that IRF2 may negatively regulated KPNA2 as its upstream TF to modulate OS progression.

This study aimed to investigate the role of IRF2 in modulating KPNA2 expression, which may serve an important role in p65 nuclear importation in the progression of OS. Here, we found that KPNA2 deficiency suppressed the malignant behaviors of OS cells, and that the underlying mechanisms involved were regulated by IRF2 and associated with EMT progress. Taken together, our findings demonstrated that KPNA2 may serve as a new potential prognostic indicator and therapeutic target for OS.

## 2. Materials and Methods

### 2.1. Cell Culture

Four human OS cell lines (Saos-2, HOS, U2OS, and MG-63) and human fetal osteoblasts cell line (hFOB 1.19) were obtained from the Cell Bank of Type Culture Collection of the Chinese Academy of Sciences (Shanghai, China). Cells were grown in Dulbecco's modified Eagle medium (DMEM; Gibco) harboring with 10% fetal bovine serum (FBS) (Gibco). All OS cell lines were cultured in a humidified incubator under 5% CO_2_ at 37°C, while hFOB1.19 cells were grown at 34°C. Mycoplasma-microbial contamination examination and STR profiling were checked to confirm the genotypes.

### 2.2. Patients and Tissue Samples

Twenty-five paired tumor samples and their adjacent nontumor tissues from patients who had undergone surgery were obtained from Zhongshan Hospital, Fudan University. This study was approved by the Ethics Committee of Zhongshan Hospital, Fudan University (Y2014-185) according to the Declaration of Helsinki, and written informed consents were obtained from all the patients. No patients had undergone chemotherapy, radiation therapy, or other related targeted therapy before surgery. The diagnosis of OS was confirmed by at least two pathologists. All surgical tissue samples used in our study were immediately placed in liquid nitrogen and then stored at -80°C until use.

### 2.3. RT-qPCR

Total RNA was isolated from OS cell lines and tissue samples with the TRIzol™ Reagent (Invitrogen) according to the manufacturer's protocol. Complementary DNA (cDNA) synthesis was performed using the PrimeScript RT reagent kit (Takara) for mRNA expression analysis. The cDNA was applied to perform qRT-PCR assay with SYBR Premix Ex Taq kit (TaKaRa) following the protocols. The 2 − ^ΔΔCt^ method was used to analyze the difference in the level of mRNA between different groups. Primers used in this study were listed as follows: KPNA2, 5′-ATTGCAGGTGATGGCTCAGT-3′ (forward) and 5′-CTGCTCAACAGCATCTATCG-3′ (reverse); IRF2, 5′-CATGCGGCTAGACATGGGTG-3′ (forward) and: 5′-GCTTTCCTGTATGGATTGCCC-3′ (reverse); The GAPDH was used as an internal control and was detected using the following primers: 5′-AATCCCATCACCATCTTCC-3′ (forward) and 5′-AGTCCTTCCACGACCAA-3′ (reverse).

### 2.4. Lentiviral Vector Encoding shRNA Plasmids

KPNA2 cDNA was cloned into the Lenti-OE vector (Genepharma, Shanghai, China) to generate KPNA2-overexpressing lentiviral vectors. Short hairpin RNAs (shRNAs) were designed by the RiboBio Co., Ltd. (Guangzhou, China). Lentiviral vectors encoding KPNA2 and IRF2 were synthesized and packaged by Genepharma company.

### 2.5. Cell Proliferation Assay

The cell proliferation assay was performed using a Cell Counting Kit (CCK-8, Dojindo, Japan) following the manufacturer's instructions. Cells at a density of 5 × 10^3^ were added into the 96-well plate and 10 microliters of CCK-8 solution was added to each well at 1, 2, 3, 4 and 5 days at 37°C. An additional 1 h later, the absorbance at wavelength of 450 nm was measured under a microplate reader.

### 2.6. Colony Formation Assay

For the colony formation assay, 500 cells were plated into each well of a 6-well culture plate. The plates containing DMEM were incubated at 37°C for 2 weeks. After being washed with PBS for three times, cells were fixed with 4% paraformaldehyde for 10 min at room temperature, followed by staining with 0.5% crystal violet solution for another 20 min. Lastly, the visible colonies of more than 50 cells were manually counted and imaged under a microscope.

### 2.7. Transwell Invasion Assay

Cells at the density of 1 × 10^4^ were seeded into a diameter Transwell plate with 8-*μ*m pores (Sigma-Aldrich). The upper chamber of the plate was added with 50 *μ*l of Matrigel collagen and 600 *μ*L of complete DMEM was added to the lower chambers, and then the cells were incubated for 24 h. The cells on the upper layer were removed and the invasive cells were fixed with 4% formaldehyde for 20 min, and then stained with crystal violet for 15 min. Cells that had invaded the bottom surface of the filter were counted to assess the invasive capacity. Invaded cells were quantified by at least five fields of view under light microscopy (Leica) to obtain the representative images.

### 2.8. Wound Healing Assay

Cells were cultured in six-well plates. After reaching 90% confluence, a 200 *μ*l pipette tip was used to create scratch wounds in the cell monolayer. Representative images of cell migration were photographed under light microscopy (Leica) at 0 and 24 h after wounding. Migration ability was assessed by measuring changes in wound width or area with ImageJ software.

### 2.9. Western Blot Assay

The protein was lysed from tissues and cells with RIPA buffer (Thermo Fisher Scientific). Protein concentration was assessed using the bicinchoninic acid (BCA) assay kit (Thermo Fisher Scientific). Equal amounts of protein samples were separated by 12% SDS-PAGE gels and transferred to polyvinylidene difluoride (PVDF) membranes. After incubation with 5% nonfat milk for 2 h at room temperature, to hatch the blots with the primary antibodies including anti-KPNA2 (ab6036), IRF2 (ab124744), E-cadherin (ab1416), N-cadherin (ab18203), Vimentin (ab92547), and GAPDH (ab9484) overnight at 4°C, which were purchased from Abcam. Hatching of horseradish peroxidase-conjugated secondary antibodies at room temperature, the endogenous GAPDH is the internal reference protein. The protein band signals were visualized on an ECL detector (Pierce) and quantified by scanning the densitometry using ImageJ software.

### 2.10. Chromatin Immunoprecipitation (ChIP) Assay

ChIP assays were performed using a kit (Sigma-Aldrich) following the protocol provided by the manufacturer. To hatch the diluted DNA-protein complex, the antibodies of anti-IRF2 and mouse IgG (Sigma-Aldrich) were added in the presence of protein A/G beads and incubated at 4°C overnight. The RT-qPCR assay was applied to examine the ChIP DNA samples. IgG was the negative control.

### 2.11. Dual Luciferase Test

Wild (WT) and mutant (MUT) of KPNA2 were inserted into the pGL3 promoter vector, which was transfected into U2OS and MG-63 cells using Lipofectamine 2000 (Invitrogen) together with plasmid of IRF2 overexpression or empty plasmid (NC). 48 h later, luciferase activity was measured using a dual-luciferase reporter assay system (Promega) following the manufacture's protocols.

### 2.12. Tumor Xenograft Assay

All animal experiments were in accordance with the Institutional Animal Care and Use Committee Guide (IACUC) of Zhongshan Hospital, Fudan University (2018-014). The mice were placed in an environmentally controlled pathogen-free isolation facility under a 12 h light-dark cycle and food and water were freely available. Subsequently, mice were randomly divided into four groups (*n* = 3/per group). Equal number of indicated U2OS cells (5 × 105) were subcutaneously implanted into the right flank of 6-week-old female athymic nude mice. Tumors were formed and the mice were anesthetized and sacrificed 24 days after tumor inoculation. After being removed, the tumors were imaged and weighed, and the volume of tumors was monitored every 3 or 5 days and calculated as follows: Volume (mm^3^) = (length × (width^2^)/2).

### 2.13. Histological Analysis and Immunohistochemistry

Xenotransplant tumor samples were isolated and fixed in 4% paraformaldehyde. Paraffin was used to embed tumor tissues and then sectioned at a thickness of 5-*μ*m. The paraffin sections were dewaxed, hydrated, and then stained with hematoxylin and counterstained with eosin. Antigens were recovered with citrate buffer and blocked with 3% H2O2, immunohistochemistry (IHC) was performed with diluted primary KI67 antibody overnight at 4°C, followed by incubation with secondary antibody at room temperature. The slides were developed by diaminobenzidine (DAB) and stained with hematoxylin. IHC staining pictures were obtained under a light microscope. All results were determined by two pathologists who were completely blinded to the grouping.

### 2.14. Function Enrichment Analysis Based on the TARGET-OS Dataset

Gene Ontology (GO) and Kyoto Encyclopedia of Genes and Genomes (KEGG) enrichment analysis were performed on the upregulated genes in the high- and low- IRF2 or KPNA2 subgroups based on the TARGET-OS dataset, and deduced their functions by analyzing the gene set. In our work, we explored whether differentially expressed genes between these subgroups were enriched among OS-related biological functions or pathways. Significant GO biological process terms and KEGG pathways with *p* < 0.05 were collected and visualized using the R package “ggplot2” (version 4.0.3).

### 2.15. Correlation Analysis of 22 Immune Cell Infiltration with IRF2 and KPNA2

22 tumor-infiltrating immune cells (TIICs) in OS samples from the TARGET dataset were assessed by applying the deconvolution algorithm (referred to as CIBERSORT) in the osteosarcoma microenvironment. Samples with *p* < 0.05 in CIBERSORT analysis result were used in further analysis. The matrix of gene expression signatures of 22 TIICs was obtained from the CIBERSORT platform (https://cibersortx.stanford.edu) [23]. The matrix data of IRF2 and KPNA2 levels were compared with those of the signature matrix of 22 TIICs from the CIBERSORT platform to generate a proportion matrix for the 22 TIICs in OS tissues.

### 2.16. Statistical Analysis

All data obtained are expressed as the mean ± standard deviation (SD). Differences Student's *t*-test or one-way ANOVA followed by a Tukey post hoc test were used to compare data between two groups or among multiple groups. Statistical difference was analyzed using the GraphPad Prism 8.0 software. Followed by a Tukey's post hoc test.

## 3. Results

### 3.1. Overexpression of KPNA2 in the Osteosarcoma Tissues

**Figure 1:**
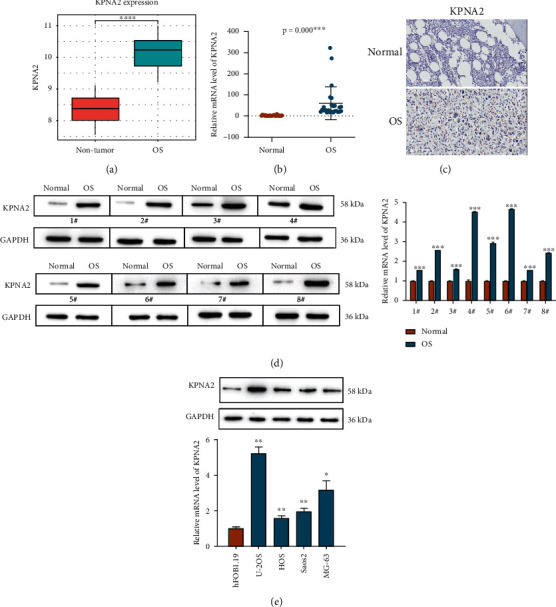
High expression of KPNA2 in osteosarcomas samples. (a) KPNA2 mRNA expression in OS and nontumor tissues based on data from GSE36001 dataset. (b) KPNA2 mRNA levels were detected in 25 pairs of OS samples and their adjacent normal samples by RT-qPCR. (c) Representative immunohistochemical (IHC) images of KPNA2 expression in OS tissues and normal tissues. (d) KPNA2 protein expression in 8 pairs of clinical OS samples were examined by western blotting. (e) KPNA2 expression in four OS cell lines and one normal hFOB1.19 cells using RT-qPCR and western blot assays. The data was presented as the mean ± SD from three independent experiments. ^∗^*p* < 0.05, ^∗∗^*p* < 0.01 and ^∗∗∗^*p* < 0.001.

Based on the GSE36001 database, we discovered KPNA2 expression was markedly upregulated in OS tissues when compared to nontumor tissues (Figure 1(a)). To examine whether KPNA2 altered clinical OS, qRT-PCR assay was performed to examine KPNA2 levels in 25 pairs of cancerous OS samples and their adjacent normal samples.  Figure 1(b) showed that the mRNA levels of KPNA2 were obviously upregulated in OS samples compared to normal samples. Representative IHC images showed similar results (Figure 1(c)). Furthermore, the protein levels of KPNA2 in 8 cases of OS samples were obviously elevated as well (Figure 1(d)). Furthermore, compared to hFOB1.19 cells, a higher level of KPNA2 in four OS cell lines, including U2OS, HOS, Saos2, and MG-63, was observed (Figure 1(e)). KPNA2 expression was the highest in U2OS and MG-63, which were chosen to use for subsequent analysis. These results suggest that KPNA2 might play a vital role in the progression of OS.

### 3.2. The Transcription Factor IRF2 Specifically Regulates the Expression of KPNA2 in Osteosarcomas

**Figure 2:**
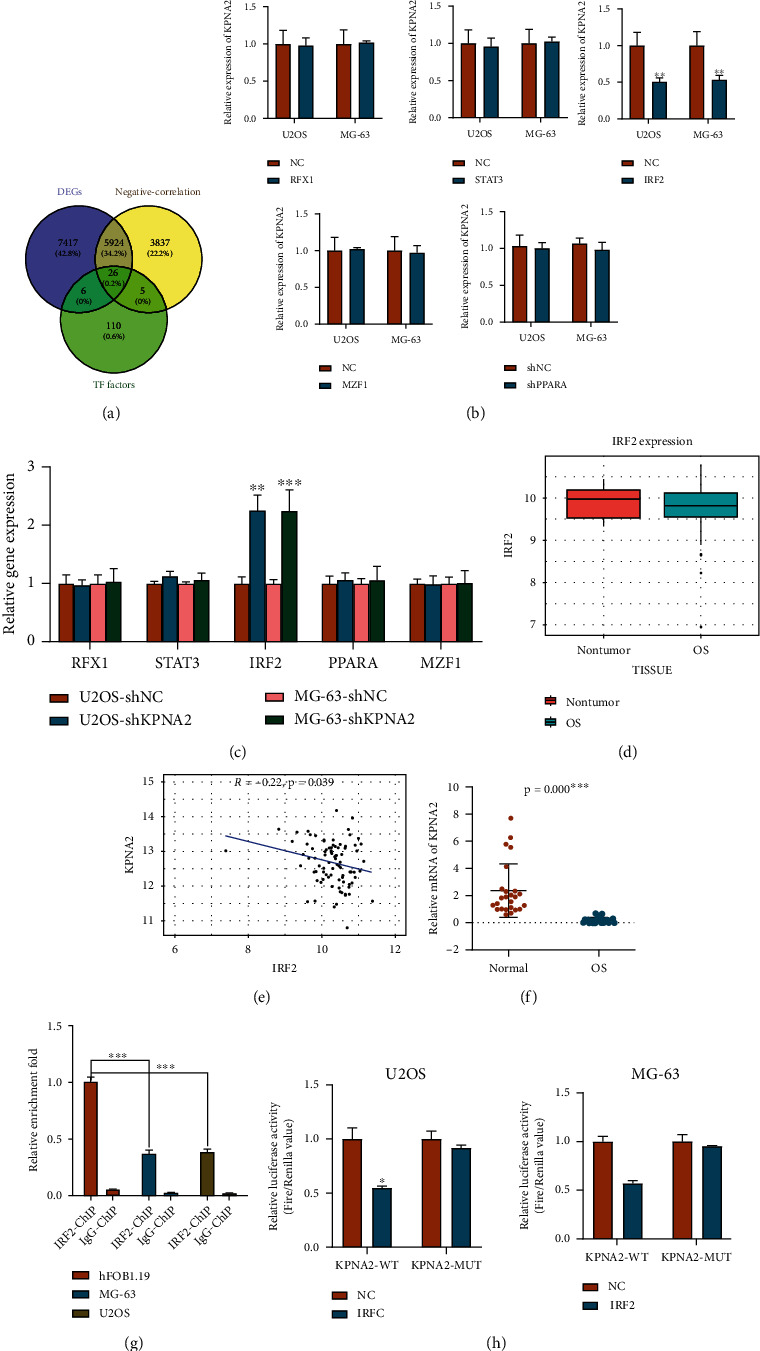
Identification of KPNA2 transcription factor (TF) (a) Venn diagram displayed the overlap among differentially expressed protein-coding genes (DEGs) from GSE157322, KPNA2 negative-related genes from TARGET dataset and TF factors of KPNA2 from online TFBIND dataset. (b) The mRNA expression of KPNA2 in response to overexpression of five candidate genes (RFX1, STAT3, IRF2, PPARA, and MZF1) in U2OS and MG-63 cells. (c) The mRNA expression of five candidate genes (RFX1, STAT3, IRF2, PPARA, and MZF1) in response to KPNA2 silence in U2OS and MG-63 cells. (d) Expression of IRF2 mRNA in OS and nontumor tissues based on data from GSE36001 dataset. (e) The negatively correlation between KPNA2 and IRF2 was analyzed under a Pearson correlation analysis according to TARGET database. (f) IRF2 mRNA expression in 25 pairs of OS and normal samples. (g) The enrichment of IRF2 bind on the KPNA2 promoter was significantly reduced in two OS cell lines when compared to hFOB1.19 cells from ChIP and qRT-PCR assays. (h) Luciferase assays were performed to detect the luciferase activity of KPNA2-WT and KPNA2-MUT after IRF2 overexpression in U2OS and MG-63 cells. The data was presented as the mean ± SD from three independent experiments ^∗^*p* < 0.05, ^∗∗^*p* < 0.01, and ^∗∗∗^*p* < 0.001.

To better understand the molecular mechanism of KPNA2 in OS, we first applied the online databases to seek KPNA2-related factors. Considering that the regulation of KPNA2 in OS was focused on the transcriptional level, we performed a bioinformatic analysis to find a transcription factor (TF) in the KPNA2 promoter region. Then, we extracted 147 KPNA2 TFs from online TFBIND dataset. Data from TARGET datasets showed that a total of 9792 genes were negatively associated with KPNA2 in OS (*R* > 0.2, FDR < 0.05). We further found that 5950 genes overlapped in downregulated gens from the GSE157322 dataset, and 31 genes were overlapped in 147 KPNA2 TFs. Therefore, a total of 26 genes were at the intersection of three (Figure 2(a)). Under the same conditions, a total of 382 genes were positively correlated with KPNA2 in OS. After the intersection of three gene sets, only one gene, MYB, was located in the center circle (Figure S1(a)). However, subsequent experiments showed that MYB was not differentially expressed in OS and was not regulated by KPNA2 (Figure S1(b) and 1(c)).

According to the correlation index of these 26 genes with KPNA2, we have chosen the top 5 TFs of KPNA2, including RFX1, STAT3, IRF2, PPARA, and MZF1. To assess whether these five TFs could alter KPNA2 expression, we overexpressed these TFs in two OS cell lines. As shown in Figure 2(b), these results demonstrated that only IRF2 overexpression significantly suppressed KPNA2 expression in two OS cell lines, while the other four TFs had no significant effect on KPNA2 expression. Meanwhile, due to the fact that KPNA2 was overexpressed in four OS cell lines, we also determined the change of these five TFs after KPNA2 knockdown using RT-qPCR. The silence of KPNA2 markedly upregulated the mRNA levels of IRF2, while the other four TFs did not obviously changed response to the elimination of KPNA2 (Figure 2(c)). Therefore, we decided to use IRF2 for subsequent experiments.

Through the GSE36001 dataset, IRF2 expression was mildly downregulated in OS tissues without significant differences (Figure 2(d)), while IRF2 expression was strongly negatively correlated with KPNA2 (Figure 2(e)). Consistently, the mRNA level of IRF2 was significantly downregulated in 25 cases of clinical OS tissue samples when compared with adjacent normal tissues (Figure 2(f)). Besides, a ChIP test was conducted in two OS cell lines and hFOB1.19 to evaluate the binding relationship of IRF2 with KPNA2, and we found that the enrichment of IRF2 binding was prominently decreased in two OS cell lines comparing to the hFOB1.19 cells (Figure 2(g)). The present findings showed that IRF2 might be one of the major regulators to regulate KPNA2 expression in OS. To further determine whether IRF2 bound to the KPNA2 promoter, a dual luciferase assay revealed that IRF2 was able to dramatically reduce the luciferase activity of KPNA2-WT, but not in KPNA2-MUT (Figure 2(h)). Collectively, IRF2, as a functional TF, could bind to KPNA2 in OS cells.

### 3.3. IRF2 Deficiency May Cooperate with KPNA2 to Regulate Cell Proliferation and Tumor Growth of OS Cells In Vivo and In Vitro

**Figure 3:**
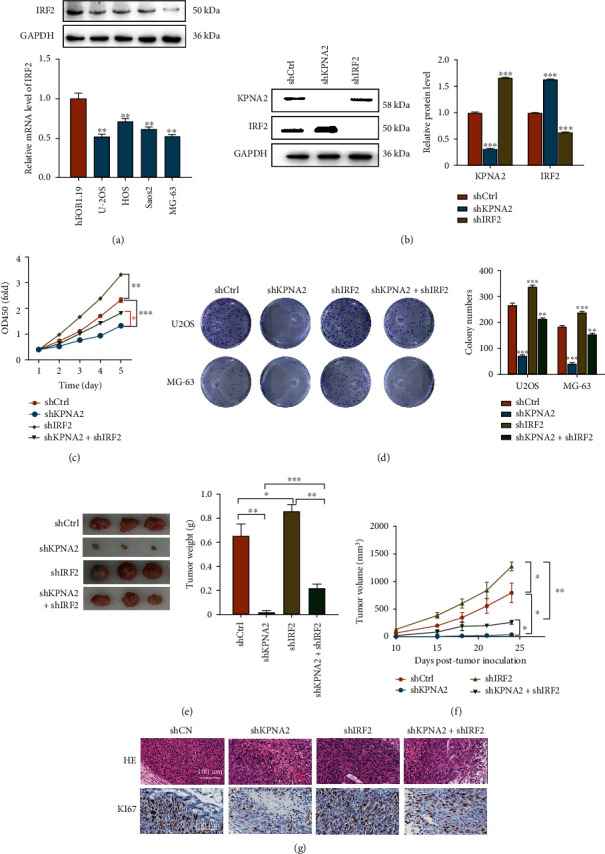
Effects of IRF2 and KPNA2 on cell proliferation, colony formation, and tumor growth of OS cells in vitro and in vivo. (a) Western blot and RT-qPCR analyses were performed to measure the relative mRNA and protein levels of IRF2 in four OS cell lines including U2OS, HOS, Saos2, and MG-63 control to the hFOB1.19 cells. (b) U2OS cells were cotransfected with shKPNA2 or shIRF2 plasmids, and the protein expression of KPNA2 and IRF2 was assessed using western blotting assay. (c) Cell viability was determined using an CCK-8 kit at different time points. (d) Cells were seeded in plates and grown for 14 days. (Left) Cell colonies were stained with 0.1% crystal violet. (Right) Colony numbers were quantified. (e–f) In vivo tumor growth. U2OS cells were injected into mice and tumor weight (e) and tumor volumes (f) were measured every 5 or 3 days for 24 days posttumor inoculation. *n* = 3. (g) Tumor tissues were separated from mice for HE and IHC staining for detecting KI67 expression. Scale bar = 100um. The data was presented as the mean ± SD from three independent experiments ^∗^*p* < 0.05, ^∗∗^*p* < 0.01, and ^∗∗∗^*p* < 0.001.

Since a negative correlation existed among KPNA2 and IRF2, we investigate their effects on cell proliferation, migration, invasion, and cell cycle. Firstly, in four OS cell lines, IRF2 protein and mRNA levels were lower than that in hFOB1.19 cells (Figure 3(a)). Downregulation of IRF2 or KPNA2 in U2OS cells was achieved by lentiviral transductions of IRF2 or KPNA2 knockdown vectors (shKPNA2 and shIRF2), as confirmed by western blot (Figure 3(b)). Regarding the malignant phenotypes of OS cells, KPNA2 knockdown inhibited cell viability, proliferation while IRF2 knockdown had the opposite effects and partially rescued above-mentioned malignant phenotypes suppressed by KPNA2 knockdown in vitro (Figures 3(c)–3(d)). In vivo, KPNA2 knockdown markedly reduced tumor weight and tumor volumes while IRF2 knockdown promoted tumor growth, and IRF2 knockdown could weaken KPNA2 knockdown-medicated tumor growth inhibition (Figures 3(e) and 3(f)). The KI67 expression was reduced by KPNA2 knockdown while elevated by IRF2 knockdown (Figure 3(g)). These findings demonstrated that IRF2 silence partially attenuates the impact of KPNA2 knockdown on OS growth.

### 3.4. IRF2/KPNA2 Might Regulate Migration and Invasion of Osteosarcoma Cells, as Well as Regulate EMT Process

**Figure 4:**
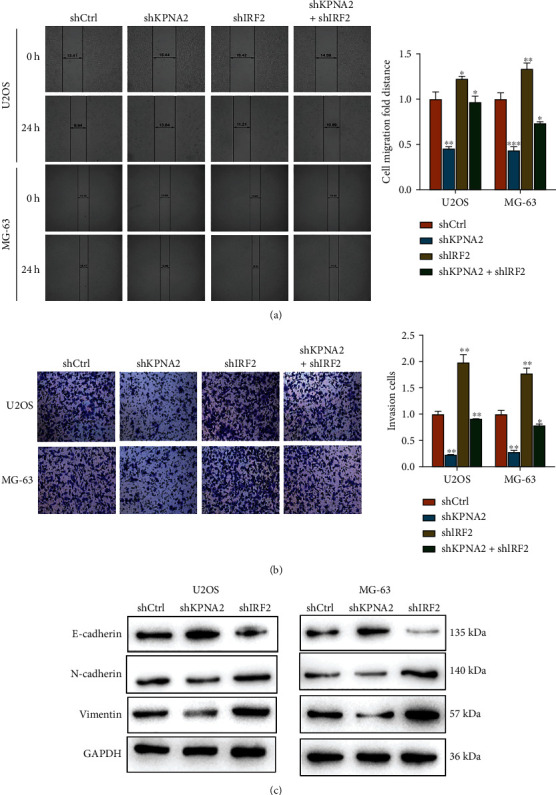
KPNA2 promoted the activation of EMT through IRF2. (a) The cell migration capacity of OS cells was examined using wound healing assays at 0 and 24 h. (b) Cells were stained with 0.1% crystal violet (left) Bars = 50 *μ*m. Cell numbers were quantified to value the invasive capacity of OS cells after different transfection (right). (c) Protein levels of EMT-related proteins (E-cadherin, N-cadherin, and Vimentin) in two OS cells (MG-63 and U2OS). Data were presented as mean ± SD from three independent experiments ^∗^*p* < 0.05, ^∗∗^*p* < 0.01, and ^∗∗∗^*p* < 0.001.

Regarding the malignant phenotypes of migration and invasion, KPNA2 knockdown inhibited the abilities of migration and invasion while IRF2 knockdown had the opposite effects and partially rescued above-mentioned malignant phenotypes suppressed by KPNA2 knockdown OS cells (Figure 4(a) and 4(b)). To investigate whether IRF2/KPNA2 expression is correlated with other molecular alterations in OS, we evaluated several molecules that are associated with EMT in tumor progression [24]. Then, the EMT-related proteins (E-cadherin, N-cadherin, and Vimentin) were detected by Western blotting. As shown in Figure 4(c), shKPNA2 significantly elevated E-cadherin while reduced N-cadherin and Vimentin expression in both U2OS and MG-63 cells. In contrast, shIRF2 had the opposite effect on these EMT-related proteins. These findings indicated that knockdown of KPNA2 may inhibite EMT progress while IRF2 silence promoted EMT progress of OS cells.

### 3.5. Functions and Pathway Enrichments Related to KPNA2 and IRF2

**Figure 5:**
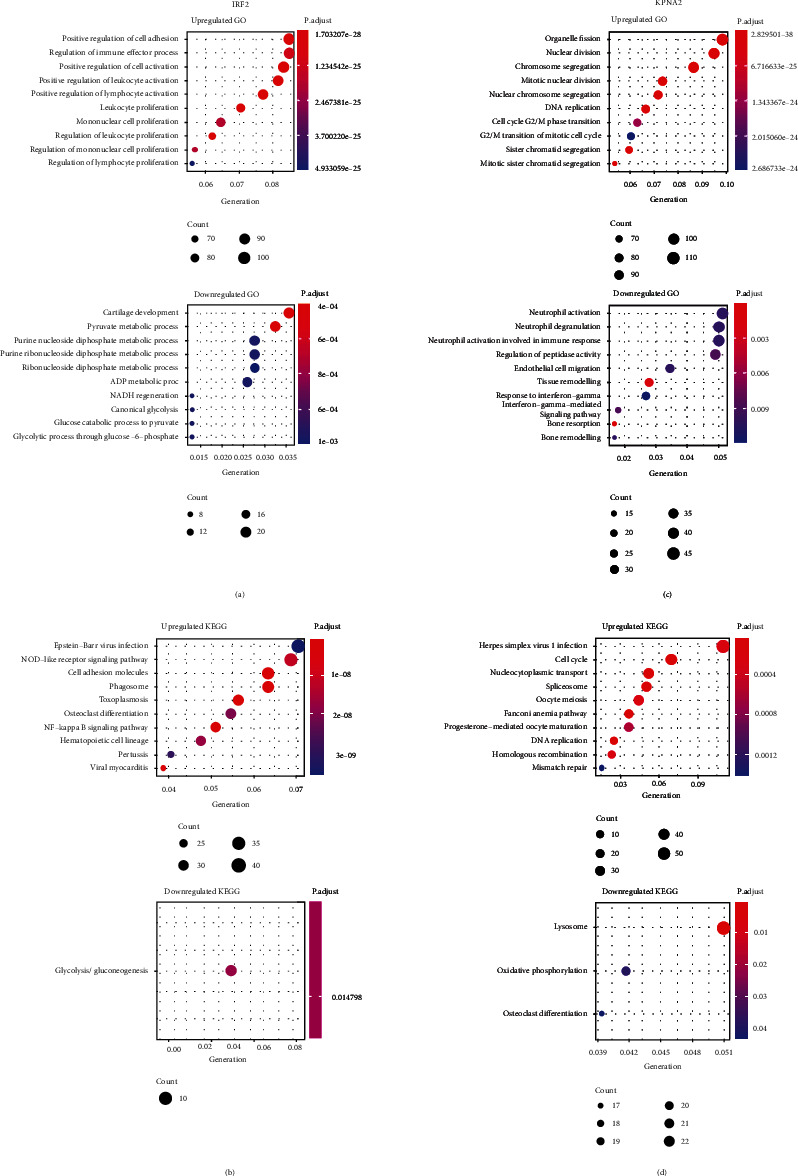
Functions and Pathway enrichments related to KPNA2 and IRF2. (a) Dotplot of the top 10 enriched terms across the upregulated-genes in the high-IRF2 and low-IRF2 subgroups using GO cluster analysis, colored according to *p*-value. (b) Dotplot of enriched pathways of upregulated genes in the high-IRF2 and low-IRF2 subgroups using KEGG analysis, colored according to *p*-value. (c) Dotplot of the top 10 enriched terms across the upregulated-genes in the high-KPNA2 and low-KPNA2 subgroups using GO cluster analysis, colored according to *p*-value. (d) Dotplot of enriched pathways of the upregulated genes in the high-KPNA2 and low-KPNA2 subgroups using KEGG analysis, colored according to *p*-value.

GO and KEGG enrichment analysis of the upregulated genes in the high- and low-IRF2 subgroups were performed. The results of the GO enrichment analysis showed that the upregulated genes in the high-IRF2 subgroup mainly played a major role in several biological processes, such as the positive regulation of cell adhesion and the regulation of the immune effector process (Figure 5(a)). The results of the KEGG enrichment analysis showed that these upregulated genes were involved in osteoclast differentiation and the NF-kappa B signaling pathway and others. (Figure 5(b)). Similarly, GO analysis revealed that several hallmarks of the tumor were enriched in the high-KPNA2 subgroup, such as DNA replication and cell cycle G2/M phase transition, while genes in the low KPNA2 subgroup was associated with immune response, bone resorption and bone remodeling (Figure 5(c)). KEGG showed that KPNA2 was related to cell cycle, DNA replication, and osteoclast differentiation (Figure 5(d)). These results are derived from the enrichment of upregulated genes related to IRF2 and KPNA2 based on the TARGET dataset, which is helpful for researchers to find possible research directions when studying the functions of IRF2/KPMA2 in OS progression.

### 3.6. The Key Infiltrating Immune Cell Related to IRF2/KPMA2 in the Osteosarcoma Microenvironment

**Figure 6:**
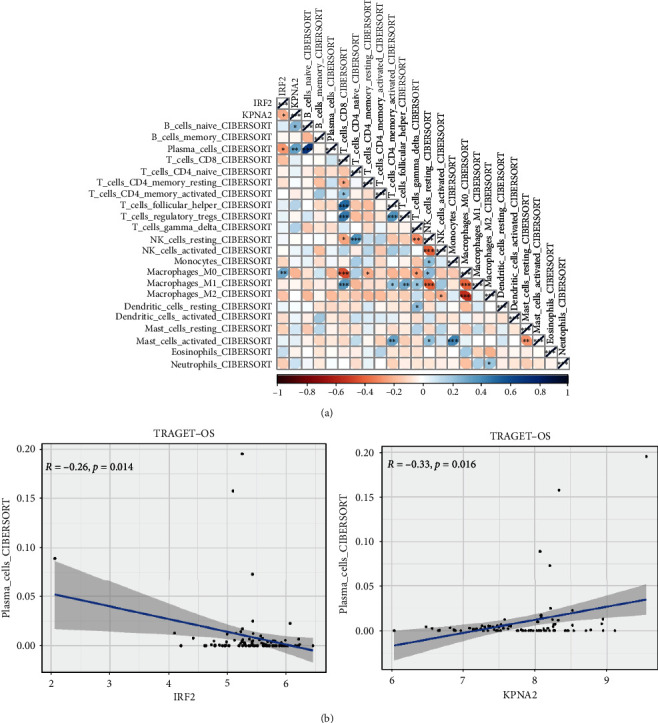
The key infiltrating immune cell related to IRF2/KPMA2 in the osteosarcoma microenvironment. (a) Correlation matrix of all 22 TIICs proportions with IRF2 and KPNA2. (b) Correlation scatter plots of plasma cell level with IRF2 and KPNA2 based on the TARGET-OS dataset using Pearson's correlation analysis.

Based on the TARGET dataset, all OS samples with low and high immune score, respectively, were eligible for CIBERSORT (*p* < 0.05). The correlations among the 22 TIICs ranged from weak to moderate. Obviously, plasma cells showed highly negative correlations with IRF2 expression, while positive correlations with KPNA2 expression (Figure 6(a)). Furthermore, the Pearson correlation scatter plot further also presented these findings (Figure 6(b)). These data suggested that plasma cells might play a key role in the IRF2/KPNA2-mediated osteosarcoma-immune interaction.

## 4. Discussion

Osteosarcomas are relatively rare but devastating [5]. Unfortunately, the introduction of new adjuvant chemotherapy after aggressive surgical resection has temporarily improved overall 10-year survival but has not significantly improved patient survival since the 1990s [25]. Therefore, it is of great significance to identify new molecules, which further helps to develop effective methods to diagnose and treat this malignant bone tumor. Here, we propose a mechanism for the role of KPNA2 in OS pathogenesis: KPNA2 may transport IRF2 into the nucleus where it regulates transitivity, triggering EMT and subsequent malignant biological properties of OS cells (Supplementary material, Graphical Abstract). This evidence may provide new ideas for the diagnosis and treatment of osteosarcoma.

Recently, several studies have linked KPNA2 to various cancers, such as lung, breast, colon, and pulmonary cancer. High KPNA2 was positively related to cancer invasiveness and poor prognosis, thus regarded KPNA2 as a potentially relevant therapeutic target for patients with different cancers [26]. KPNA2 was involved in several cellular biological processes, including cell differentiation, development, viral infection, immune response, and transcriptional regulation [6]. Similarly, our study illustrated that KPNA2 was dramatically elevated in OS samples compared to normal samples. Although KPNA2 has been shown to be frequently expressed in OS as a new marker for the diagnosis, as well as in chondrosarcoma and Ewing sarcomas [27], the functions of KPNA2 in osteosarcoma are unclear. In the present study, data mining and bioinformatics analysis indicated that KPNA2 was overexpressed in OS patients from GSE36001 dataset, and the experiments verified high KPNA2 level in clinical OS samples and OS cell lines. Furthermore, KPNA2 knockdown inhibited the proliferation, migration, and invasion in two OS cell lines, and remarkably reduced tumor weight and tumor volumes in vivo. These findings revealed that KPNA2 might play a crucial role in the biological progress of OS.

Interferon regulatory factor-2 (IRF2) exerted antitumor effects in several human cancers. For example, IRF2 could suppress cell proliferation and migration ability and promote cell apoptosis in nonsmall cell lung cancer cells [28]. IRF2 might play as a tumor suppressor by regulating P53 signaling in gastric cancer [29]. Furthermore, IRF2 was shown to serve as a tumor suppressor in patients with hepatocellular carcinoma, whose inactivation led to impaired TP53 function [30]. The current study highlighted that KPNA2 could negatively alter IRF2 expression in OS cells. Meanwhile, by data mining in the GSE157322 and TARGET datasets, we discovered that IRF2 could bind to the KPNA2 promoter and activate KPNA2 expression by bioinformatic analysis for TF prediction. This underlying mechanism was consistent with a previous report that IRF2 could bind to the miR-1227 promoter, thus inhibiting tumor growth [21]. Furthermore, IRF2 was obviously downregulated, which was negatively associated with KPNA2 in OS. More importantly, IRF2 knockdown promoted malignant behaviors, which were seemingly suppressed by KPNA2 knockdown. These rescuing effects of IRF2 on KPNA2 were also reflected in tumor growth in vivo. These findings demonstrated that IRF2 silence might partially attenuate the impact of KPNA2 knock-down on osteosarcoma progressions.

EMT is regulated by various signaling pathways, including NF-*κ*B, Wnt, and transforming growth factor-*β* [13]. In our study, the key outcome is that migration and invasion of OS cells were significantly inhibited by deletion of KPNA2, which also resulted in a decrease in the EMT characteristics of OS cells; epithelial cell markers were increased, and mesenchymal markers were decreased. However, the opposite results were found after IRF2 was knocked down. KPNA2 and IRF2 have opposite regulatory effects on the activation of EMT progress of OS cells. Thus, KPNA2 might contribute to the progression of OS by negatively regulating IRF2 via promoting EMT.

KPNA2 is involved in the nucleocytoplasmic transport pathway of multiple tumor-associated proteins and is overexpressed in various cancers thereby being suggested as a prospect in the diagnosis and treatment of cancer [6]. Given that IRF2 has the ability to exert antioncogenic activities, IRF2 overexpression led to a dramatic cell death response by apoptosis in hepatocellular carcinoma [31]. These are consistent with our results of functional enrichment, showing these genes are related to DNA replication and cell cycle processes. Furthermore, it could be observed that KPNA2 was positively correlated with plasma cell level, while IRF2 had a negative relationship with plasma cell level. Infiltrating immune cell subsets detected by CIBERSORT analysis can reflect the time course of innate and adaptive immune responses in OS. CIBERSORT may have the potential to characterize the detail of infiltrating immune cells in OS tissues and provide novel insights into the pathogenesis of OS. To our knowledge, these two genes are the first to link immune cell levels in the osteosarcoma microenvironment in this study, with the hope of providing guidance for the next study of relevant molecular mechanisms.

However, most of the experiments in our study are performed using isolated tumor cell lines cultured in vitro or immunodeficient nude mice with human U2OS xenografts, which do not account for any tumor-extrinsic effects that the candidate signaling pathway may have on immune cell interactions with tumor cells. This limitation suggests that studying tumor-immune interactions will be a potential future direction that will extend from this study.

Taken together, our results illustrated that KPNA2 regulated OS development, as well as IRF2 play a potential upstream TF of KPNA2 in regulating EMT progress. This may provide a novel target for OS therapy. Therefore, the treatment of OS with restraint of KPNA2 or IRF2 overexpression may be extra to other therapeutic interventions for the development of this disease.

## Data Availability

All data in the results of this study can be obtained on a reasonable request from the corresponding authors. Public data sources include GSE36001, GSE157322, and TARGET database online.

## Supplementary Materials

Supplementary Materials Supplementary Figure 1. Identification of KPNA2 transcription factor (TF). (a) Venn diagram displayed the overlap among differentially expressed protein-coding genes (DEGs) from GSE157322, KPNA2 positive-related genes from TARGET dataset and TF factors of KPNA2 from online TFBIND dataset. (b) Expression of IRF2 mRNA in OS and nontumor tissues based on data from the GSE36001 dataset. (c) The mRNA expression of MYB in response to KPNA2 silence in U2OS and MG-63 cells. Data were presented as mean ± SD from three independent experiments. ^∗^*p* < 0.05, ^∗∗^*p* < 0.01 and ^∗∗∗^*p* < 0.001.
