# Supplementary figures and images for: IRF2 Destabilizes Oncogenic KPNA2 to Modulate the Development of Osteosarcoma
Source: J Oncol. 2022 Sep 26;2022:9973519. doi: 10.1155/2022/9973519 (PMC9529396; doi:10.1155/2022/9973519)

A

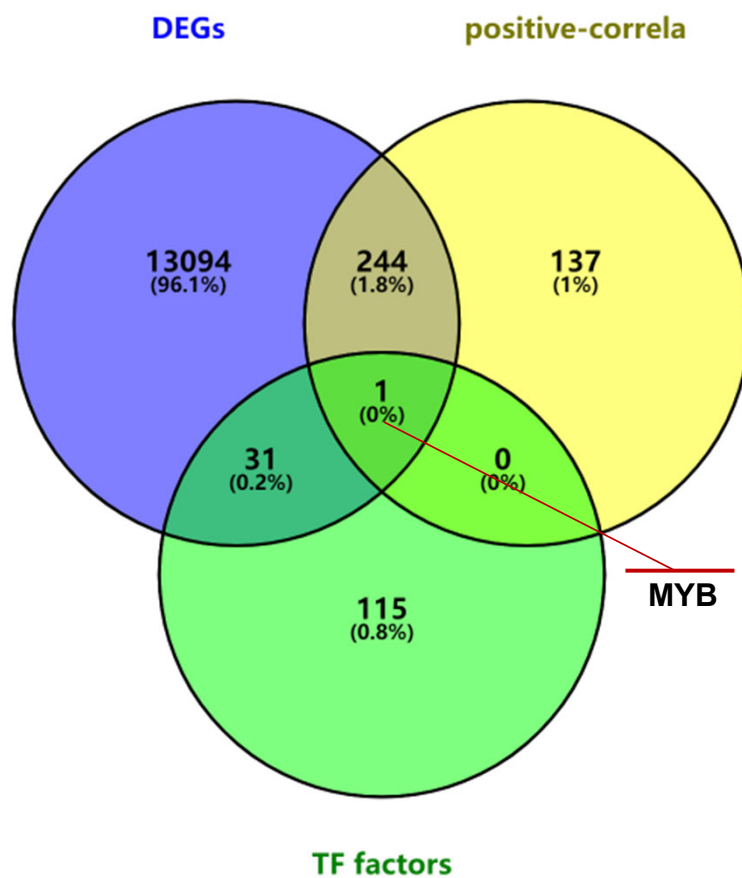

B

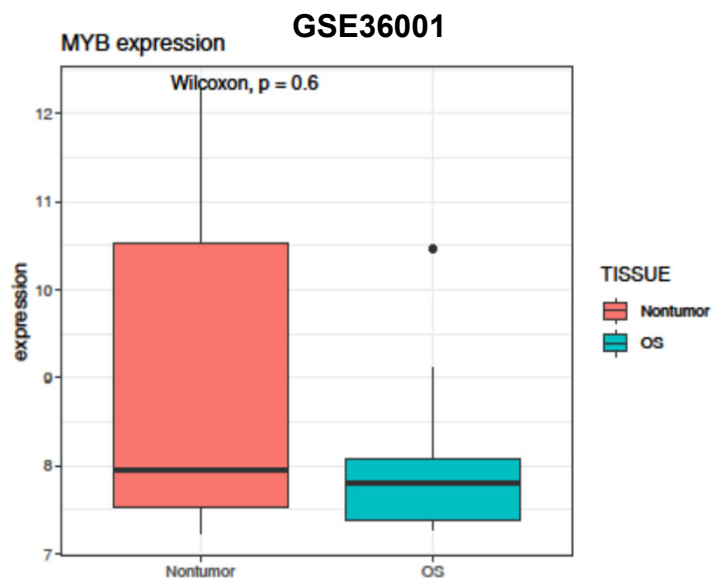

C

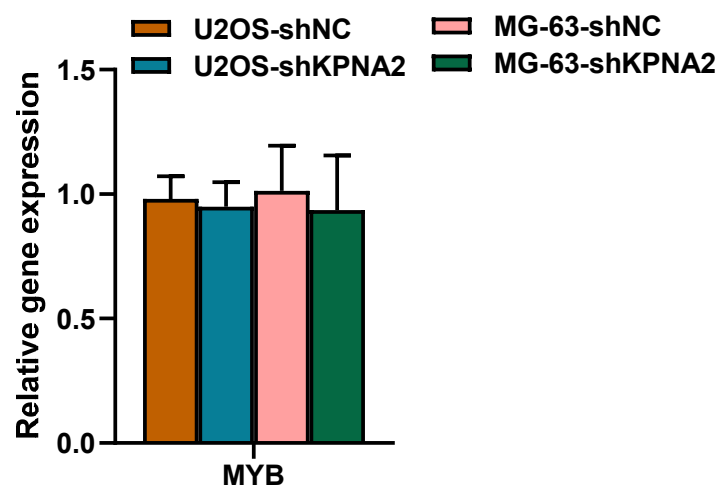

Supplement: Supplementary Materials — Supplementary Figure 1. Identification of KPNA2 transcription factor (TF). (a) Venn diagram displayed the overlap among differentially expressed protein-coding genes (DEGs) from GSE157322, KPNA2 positive-related genes from TARGET dataset and TF factors of KPNA2 from online TFBIND dataset. (b) Expression of IRF2 mRNA in OS and nontumor tissues based on data from the GSE36001 dataset. (c) The mRNA expression of MYB in response to KPNA2 silence in U2OS and MG-63 cells. Data were presented as mean ± SD from three independent experiments. ∗p < 0.05, ∗∗p < 0.01 and ∗∗∗p < 0.001. [file 9973519.f1.pdf]
